# Vitamin D Status and VDR Genotype in NF1 Patients: A Case-Control Study from Southern Brazil

**DOI:** 10.1155/2015/402838

**Published:** 2015-06-16

**Authors:** Larissa Souza Mario Bueno, Clévia Rosset, Ernestina Aguiar, Fernando de Souza Pereira, Patrícia Izetti Ribeiro, Rosana Scalco, Camila Matzenbacher Bittar, Cristina Brinckmann Oliveira Netto, Guilherme Gischkow Rucatti, José Artur Chies, Suzi Alves Camey, Patricia Ashton-Prolla

**Affiliations:** ^1^Laboratório de Medicina Genômica, Centro de Pesquisa Experimental, Hospital de Clínicas de Porto Alegre (HCPA), Porto Alegre, RS, Brazil; ^2^Programa de Pós Graduação em Medicina: Ciências Médicas, Universidade Federal do Rio Grande do Sul (UFRGS), Porto Alegre, RS, Brazil; ^3^Universidade Vila Velha, Vila Velha, ES, Brazil; ^4^Hospital Metropolitano, Serra, ES, Brazil; ^5^Departamento de Genética, UFRGS, Porto Alegre, RS, Brazil; ^6^Laboratório de Patologia Clínica, Hospital de Clínicas de Porto Alegre (HCPA), Porto Alegre, RS, Brazil; ^7^Serviço de Genética Médica, Hospital de Clínicas de Porto Alegre (HCPA), Porto Alegre, RS, Brazil; ^8^Laboratório de Imunogenética, UFRGS, Porto Alegre, RS, Brazil; ^9^Departamento de Estatística, UFRGS, Porto Alegre, RS, Brazil

## Abstract

Neurofibromatosis type 1 (NF1) patients are more likely to have vitamin D deficiency when compared to the general population. This study aimed to determine the levels of 25-OH-vitamin D [25(OH)D] in individuals with NF1 and disease-unaffected controls and analyze *Fok*I and *Bsm*I *VDR* gene polymorphisms in a case and in a control group. Vitamin D levels were compared between a group of 45 NF1 patients from Southern Brazil and 45 healthy controls matched by sex, skin type, and age. Genotypic and allelic frequencies of *VDR* gene polymorphisms were obtained from the same NF1 patients and 150 healthy controls. 25(OH)D deficiency or insufficiency was not more frequent in NF1 patients than in controls (*p* = 0.074). We also did not observe an association between *Fok*I and *Bsm*I *VDR* gene polymorphisms and vitamin D levels in NF1 patients, suggesting that their deficient or insufficient biochemical phenotypes are not associated with these genetic variants. The differences between the groups in genotypic and allelic frequencies for *Fok*I and *Bsm*I *VDR* gene polymorphisms were small and did not reach statistical significance. These polymorphisms are in partial linkage disequilibrium and the haplotype frequencies also did not differ in a significant way between the two groups (*p* = 0.613).

## 1. Introduction

Neurofibromatosis type 1 (NF1) is an autosomal dominant disease caused by mutations in the* NF1* gene, mapped at chromosome 17q11.2, which produces an ubiquitous protein called neurofibromin. NF1 is a cancer predisposition disease with variable expressivity. The main features involve the skin, bone, and central nervous system. Approximately one-half of the cases are familiar and the remainder are caused by de novo mutations in the* NF1* gene. The estimated incidence of the disease is 1 in 2.500–3.500 live births, independent of gender and ethnic background [1–5]. The diagnosis of NF1 is usually clinical and most of the affected individuals are identified in infancy or childhood. The clinical diagnosis is made when at least two of the National Institute of Health (NIH) Diagnostic Criteria for NF1 are met [6]. One of these criteria is skeletal lesions, such as sphenoid dysplasia or thinning of the long bone cortex with or without pseudoarthrosis. In addition to the classical signs and symptoms involving skeleton, NF1 patients are prone to osteomalacia, osteopenia, and osteoporosis of unknown etiology [7–12]. Neurofibromin functions as a GTPase in mesothelial-derived tissues including blood cells, fibroblasts, and osteoprogenitor cells, leading to deregulation of osteoblast and osteoclast activity [13]. However, bone constitution depends on the density and also mineral content of the bone. Therefore, metabolic abnormalities may also contribute to a predilection for bone defects in NF1, like bone-regulating hormones (i.e., vitamin D). A few studies have suggested that NF1 patients are more commonly diagnosed with hypovitaminosis D when compared to the general population [9–12, 14, 15].

Vitamin D plays a pivotal role in the homeostasis of body calcium. It increases the absorption of calcium from the small intestine and promotes its reabsorption back into bones, an essential process for proper bone metabolism. Ultraviolet B light photoisomerizes provitamin D to vitamin D in the skin, which is transported to the liver and hydrolyzed to 25-hydroxy-vitamin D [25(OH)D]. Further hydroxylation of 25(OH)D to 1.25-dihydroxy-vitamin D [1.25(OH)_2_D], the physiologically active form of vitamin D, occurs mainly in the kidney. In the clinic, 25(OH)D levels are used to assess vitamin D status since 1.25(OH)_2_D usually reflects serum calcium better than total vitamin D content. Several factors interfere with serum vitamin D levels such as age, sun exposure, skin type [17], and disorders that interfere with vitamin D metabolism (hepatic, kidney, and intestinal disease). Vitamin D insufficiency is associated with osteoporosis, bone fractures, decreased immune function, bone pain, and muscle weakness and possibly with propensity to cancer and cardiovascular disease [18–21]. 1.25(OH)_2_D exerts its biological effects through binding to the vitamin D receptor (VDR), a nuclear receptor that acts as a transcription factor. Calcium absorption occurs primarily in the duodenum where the VDR is expressed in the highest concentration, so the regulation of* VDR* gene is most important in high efficiency of calcium absorption [22]. Vitamin D receptor's genotypes have been associated with the development of several bone diseases as well as multiple sclerosis (MS), osteoporosis, and vitamin D-dependent rickets type II and other complex maladies [23].

The gene encoding the VDR is mapped on the long arm of chromosome 12 (12q12–14) and is composed of 9 exons, with an alternatively spliced promoter region [24]. A series of polymorphisms in the* VDR* gene were reported to be linked to various biological processes [25]. FokI restriction enzyme can identify a variable site in exon 2 of the gene. This alteration is characterized by a C/T transition located inside a start codon (ATG), and when the C variant is present, an alternative start site is used, leading to the expression of a shorter VDR protein (424aa), which demonstrates increased biological activity compared to the longer one (427aa) [26].* Bsm*I polymorphism apparently does not change the translated protein [25]. This G/A polymorphism is located on intron 8 and is linked in a haplotype with variable-length poly A sequence within the 3′ untranslated region, altering VDR mRNA stability [27]. Therefore, presence of both* Fok*I and* Bsm*I polymorphisms can result in decreased VDR receptor expression. We hypothesized that since VDR receptor mediates the effects of 1.25(OH)_2_D, its reduced expression may also reduce 1.25(OH)_2_D activity, even when normal vitamin D levels are present. This mechanism would affect vitamin D activity. Low vitamin D levels or decreased vitamin D activity could impair calcium absorption in duodenum and consequently, the lack of calcium could decrease bone turnover. This alteration in bone metabolism may not be sufficient to cause the classical signs and symptoms involving the skeleton in NF1 patients but may have an association that influences their occurrence, acting together with deregulation of osteoblast and osteoclast activity. Differences in* VDR* allele frequencies for* Fok*I and* Bsm*I polymorphisms between NF1 patients and the general population or differences in vitamin D levels between groups could help to clarify this possible association.

Therefore, the aim of this study was to assess and compare 25(OH)D levels in a group of 45 patients with the clinical diagnosis of NF1 with 45 sex-, skin type-, and age-matched controls' group. We sought to correlate clinical features of NF1 with serum vitamin D levels and to investigate whether* Fok*I and* Bsm*I polymorphisms in the* VDR* gene were associated with hypovitaminosis D and the NF1 phenotype. Secondly, we compared genotypic and allelic frequencies of* Fok*I and* Bsm*I polymorphisms in the* VDR* gene between NF1 group and a control group.

## 2. Materials and Methods

### 2.1. Vitamin D Status

#### 2.1.1. Patients and Controls

A consecutive series of NF1 patients seen at the genetics outpatient clinics of Hospital de Clínicas de Porto Alegre (HCPA), Southern Brazil (30° 2′ 0′′ south, 51° 12′ 0′′ west), from November 18 to December 20, 2009, were invited to participate in this study and enrolled after signature of informed consent. The study was approved by the Institutional Research and Ethics Committee of HCPA. The minimum number of patients and controls to be enrolled was estimated at 22 in each group and was calculated using Winpepi version 9.2 based on the findings of Lammert et al. [14] with a power of 90% and an alpha = 0.05. Considering the possibility of differences in sun exposure between individuals from this study (recruited in the spring in Southern Brazil) and those of Lammert et al. [14] (recruited in Germany during the winter, spring, or summer) and in order to have sufficient patients to allow clinical correlations, we set the group sizes at 45 individuals each. The group of cases consisted of adult individuals (above age 18 years) diagnosed with NF1 according to the Criteria of the Consensus Development Conference [6]. Controls were recruited from the companions of patients seen in the same genetics clinics and were matched to cases by sex, type of skin, and age (allowing a difference of ±5 years at the most). Exclusion criteria for both groups were age < 18 years, incapacity to provide informed consent, vitamin D supplementation within the last 6 months, diagnosis of gastrointestinal, skin, liver, kidney, or parathyroid disease, use of medication that could interfere with the vitamin D metabolism, known vitamin D deficiency, and hospitalization in the previous 2 months. In addition, we clinically excluded NF1 patients who met criteria for other genetic disorders such as Noonan syndrome and segmental NF1 and controls with 1st, 2nd, or 3rd degree family history of NF1.

#### 2.1.2. Clinical Evaluation

Data on clinical presentation was obtained from chart review and full physical examination was performed on all participants. To model NF1 phenotype, the presence of eight major NF1 clinical features was evaluated: café-au-lait spots, cutaneous neurofibromas, plexiform neurofibromas, axillary and inguinal freckling, optic pathway glioma, Lisch nodules, sphenoid wing dysplasia, and tibial pseudoarthrosis. The numbers of café-au-lait spots and cutaneous neurofibromas were also obtained. Family history of all participants was assessed and registered in pedigrees. The clinical assessment was performed before vitamin D testing by the same clinical geneticist.

#### 2.1.3. Vitamin D Dosage

In order to limit the effect of seasonal fluctuations of vitamin D photosynthesis, patients were recruited in the spring between the dates previously described. Fasting (minimum 4 hours) peripheral blood samples were collected in EDTA and the plasma isolated by centrifugation was frozen within one hour of collection at −80°C for posterior analysis. All samples were analyzed simultaneously after a storage period of five months. Plasma 25(OH)D levels were measured by chemiluminescence using the LIAISON commercial kit (DiaSorin Inc., Stillwater/MN.CV 6% intra-assay). Samples were scored as vitamin D deficient when 25(OH)D results were <20 ng/mL. The normal cutoff for 25(OH)D levels was defined at >30 ng/mL. Plasma levels between 20 and 30 ng/mL were classified at the insufficiency status.

### 2.2. VDR Genotyping

To determine* VDR* genotype, genomic DNA from 45 NF1 patients and a healthy control group of 150 patients was extracted from leukocytes by conventional salting-out methods. Analysis of the* Fok*I (rs2228570; T and C alleles) and* Bsm*I (rs1544410; A and G alleles) polymorphisms in the* VDR* gene was performed by PCR-RFLP in duplicate as described by Monticielo et al. [28] and was blinded for vitamin D status and clinical phenotype. The control group, constituted of 150 healthy individuals, was recruited from Porto Alegre and previously tested for the* Fok*I and* Bsm*I polymorphisms with the same methodology as described above and tested in the same laboratory as the NF1 samples.

### 2.3. Statistical Analyses

All analyses were done using the statistical package SPSS version 18.0. For categorical variables the chi-square and Fisher's exact tests were used and for quantitative variables Student's *t*-test was used. A *p* value < 0.05 was considered statistically significant.

## 3. Results

**Figure 1:**
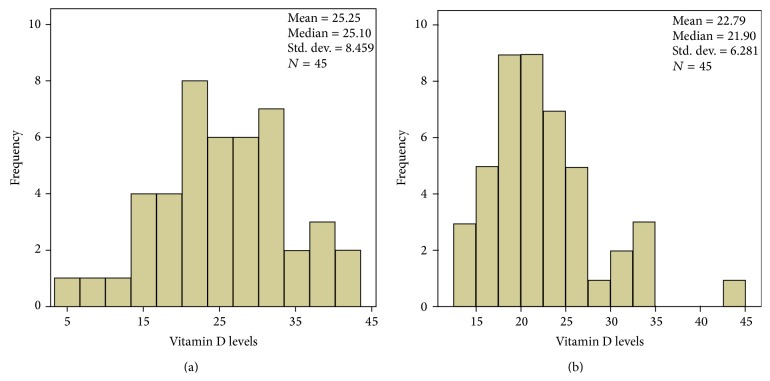
Histograms showing the distribution of plasma 25(OH)D levels (ng/mL) in NF1 patients (a) and controls (b).

Clinical and demographic features of the patients and controls used to determine vitamin D status are summarized in Tables 1 and 2. There was no significant difference between groups in age at assessment, sex, skin type (according to the Fitzpatrick classification, avoidance of sun exposure), habit of smoking, or use of alcohol. As expected, patients with NF1 had an increased frequency of short stature and had been more often diagnosed with cancer when compared to controls. The mean body mass index (BMI) for NF1 patients was 24,61 and 24,20 for controls, showing no difference between groups for this measure. The mean and median 25(OH)D levels in NF1 patients were 25.25 ng/mL and 25.10 ng/mL (±8.46), respectively, and 22.79 ng/mL and 21.90 ng/mL (±6.28) in controls, respectively. There was no statistically significant difference in mean 25(OH)D levels between the NF1 and control groups (*p* = 0.074). In the NF1 group, 29 (64.4%) of the 45 individuals studied had levels of 25(OH)D below 30 ng/mL: vitamin D deficiency was observed in 11 (24.4%) and vitamin D insufficiency in 18 (40.0%) subjects. The minimum 25(OH)D level detected in this group was 5.27 ng/mL and maximum level was 41.3 ng/mL. In the control group, 39 (86.6%) of the 45 individuals studied had levels of 25(OH)D below 30 ng/mL: vitamin D deficiency was observed in 17 (37.7%) and vitamin D insufficiency in 22 (48.8%) subjects. The minimum 25(OH)D level detected in this group was 14.1 ng/mL and maximum level was 44.3 ng/mL. When we categorized 25(OH)D using a cutoff of 30 ng/mL, NF1 patients had more frequently normal 25(OH)D levels than controls. Although this difference did not reach statistical significance, distinct distribution can be further observed in the 25(OH)D levels (ng/mL) histograms depicted in Figure 1. We did not observe a more severe phenotype in NF1 patients with lower 25(OH)D levels (data not shown).

*VDR* genotyping results of the NF1 patients are depicted in Table 3. Genotypic frequencies of the* Fok*I and* Bsm*I polymorphisms were in Hardy-Weinberg equilibrium. When compared to a subset of 150 healthy, NF1 unaffected individuals recruited at the same hospital as the NF1 patients (as described by Monticielo et al. [28]), allelic and genotypic frequencies encountered in the patients did not differ significantly. These polymorphisms are in partial linkage disequilibrium and the haplotype frequencies also do not differ in a significant way between the two groups (*p* = 0.613). Additionally, we compared 25(OH)D levels obtained from NF1 patients with their different* Fok*I and* Bsm*I genotypes (Table 4) and did not find any association.

## 4. Discussion

So far, seven studies assessed 25(OH)D levels in patients diagnosed with NF1 (Table 5). Among these, six were case-control studies and one was a descriptive study, all undertaken in the Northern Hemisphere (USA and Europe). Although biologically plausible, the association of NF1 with vitamin D deficiency remains controversial and has not been clearly demonstrated in all studies, corroborating our findings in a Southern Brazilian population. Hypovitaminosis D might indeed be involved in the pathogenesis of bone, neurological, and skin disorders of NF1, since it has a significant role in calcium homeostasis and bone metabolism but it is also involved in the regulation of cell proliferation, differentiation, apoptosis, and angiogenesis. In this line, there is consistent evidence in favor of a role for vitamin D in the expression of genes related to decreased cell proliferation for both normal and cancer cells and induction of terminal cell differentiation [19–21, 29]. However, only one group [14] described an inverse association between increased number of neurofibromas and low plasma 25(OH)D levels, suggesting an effect of the vitamin levels on disease expression. Against this hypothesis, Stevenson and colleagues [15] found no association between levels of 25(OH)D and the occurrence of optic gliomas or neurofibromas in NF1 patients. Hockett and colleagues [16] described in United Kingdom a case-control study in which the overall mean of 25(OH)D levels in control group was within deficient range and showed no statistically significant difference with NF1 group. This deficient 25(OH)D value found in control population also occurs in our control group and may reflect poor sun exposure of these populations.

In the 1990s, Nakayama and colleagues suggested an improvement of two cardinal signs of NF1, neurofibromas (NF) and café-au-lait spots (CLS) in patients treated with vitamin D [30, 31]. In addition, Yoshida et al. [32] published a paper in which eight patients with the clinical diagnosis of NF1 were treated with intense light radio frequency combined with topical vitamin D, with improvement of the phenotype. Such findings could be explained by the potent antiproliferative effect of vitamin D by inhibiting the transcription specific genes (i.e., c-fos oncogene, as observed in experimental studies with mice). Finally, Lammert et al. [14] suggested that the lower vitamin D levels observed in NF1 patients relative to controls could be related to less exposure to sunlight in patients with greater visibility of the disease. The frequency of vitamin D deficiency in the Nordic countries is higher than expected by both low sun exposure and low dietary intake of vitamin D precursors [33]. This can easily be explained by geographic and cultural aspects of those countries. In Brazil, a country with tropical and subtropical climates (depending on the geographic region), adequate 25(OH)D levels have been reported in the general population of the city of Recife (8°S) in the northeastern region. In the southeast part of the country, studies are controversial, showing normal 25(OH)D levels in the population of the State of São Paulo (21°S) but hypovitaminosis D in 42.4% in the population of Minas Gerais (19°S). In the southernmost State of Rio Grande do Sul (30°S), probably due to its climatic conditions and the risk profile of most of the individuals studied to date (hospitalized patients), a high prevalence of hypovitaminosis D has been observed [34–40]. In a cross-sectional study with resident physicians of Hospital de Clínicas de Porto Alegre (the same hospital from which the patients in this study derive), the mean serum level of 25(OH)D was 17.9 ± 8.0 ng/mL and 57.4% of them presented 25(OH)D below 20 ng/mL [38]. The high overall frequency of vitamin D deficiency and insufficiency observed in this study corroborates with previous reports that studied populations from Southern Brazil. The reasons why the overall frequency of hypovitaminosis D is so high in this study remain elusive and the lack of an observed difference between NF1 patients and controls may be related to the deficient and insufficient status of a significant proportion of individuals in the community. We can not exclude certain ascertainment biases such as the period of study (collection during the summer could definitively exclude lack of sun exposure as a factor) and acknowledge that the study has a limitation regarding sample size. However, the lower 25(OH)D levels consistently observed in controls, in terms of both mean values and distribution of individual 25(OH)D measurements, are against the hypothesis of an association of hypovitaminosis D and NF1. In addition our data, despite limited sample size, confirm previous results that differences between NF1 patients and controls are likely not major. Finally, although functional data have been inconclusive for* Bsm*1* VDR* gene polymorphism, several small studies evaluating this polymorphism have reported significant associations with osteoporosis. Some studies have shown a relationship between* VDR* polymorphisms and bone mineral density, serum 25(OH)D levels, and neoplastic and immune diseases [41–45]. Based on these articles, 65.9% of studies reported a significant correlation between* Bsm*I and osteoporosis risk. Likewise, 60.0% of studies reported a significant correlation between* Fok*I* VDR* gene polymorphism and osteoporosis risk. As expected, in our study,* VDR* gene polymorphisms* Fok*I and* Bsm*I were not more common in vitamin D deficient or insufficient NF1 patients, suggesting that these biochemical phenotypes are not related to these genetic variants. As we hypothesized,* VDR* gene polymorphisms* Fok*I and* Bsm*I could interfere in vitamin D activity, even when normal levels are present. The effects of* VDR* gene polymorphisms are in connection with each other, but the different haplotypes between the studied groups also did not reach statistical significance. The reasons for the heterogeneous results found in many association studies are numerous and varied. Sample sizes, ascertainment differences, population, and trait genetic heterogeneities may be mentioned. In addition, in quantitative characteristics, most factors account for only a small proportion of the total genetic risk.

In our patient series, the differences in vitamin D levels between cases and controls are not statistically significant; however, the lowest vitamin D levels of the series are found in NF1 patients (5 individuals with levels under 15 ng/mL). Curiously, the two patients with the lowest vitamin D levels (5.24 and 8.45 ng/mL) also have the largest number of cutaneous neurofibromas (50–100 neurofibromas), although an association between NF1 phenotype severity and lower 25(OH)D levels was not demonstrated.

## 5. Conclusion

In conclusion, there is no evidence of lower vitamin D levels in NF1 patients and no association between* VDR* gene polymorphisms and the occurrence of the disease in this group of NF1 patients from Southern Brazil. Additional studies are necessary to definitively exclude or show a role for VDR polymorphisms and vitamin D levels on the skeletal signs and symptoms of NF1.

## Figures and Tables

**Table 1:** Clinical and demographic features of NF1 patients and controls included in the study.

Features	NF1 patients (*n* = 45)		Controls (*n* = 45)		*p* value
	*N* (%)	Media/range (years)	*N* (%)	Media/range (years)	
Gender Female	31 (68.9)		33 (73.3)		0.646^3^
Age		38.6/18 to 72		36.7/18.6 to 58.6	0.212^5^
Skin type (Fitzpatrick)					0.129^4^
1	1 (2.2)		0		
2	8 (17.8)		12 (26.7)		
3	13 (28.9)		19 (42.2)		
4	5 (11.1)		7 (15.6)		
5	14 (31.1)		5 (11.1)		
6	4 (8.9)		2 (4.4)		
Habit of smoking	6 (13.3)		8 (17.8)		0.722^3^
Use of alcohol^1^	26 (57.8)		21 (46.7)		0.297^3^
No sun avoidance	39 (86.7)		35 (77.8)		0.275^3^
Previous cancer diagnosis^*∗*^	5 (11.1)		0 (0.0)		0.021^3^
Short stature^2^	11 (25.5)		3 (6.8)		0.011^3^

^1^Socially; ^2^according to the World Health Organization (*p* < 3) for controls and to Neurofibromatosis 1 Growth Charts for the cases; ^3^Fisher's exact test; ^4^chi-square test; ^5^Student's *t*-test.

^*∗*^Breast cancer (*n* = 1); Hurthle cell adenoma (*n* = 1); schwannoma (*n* = 1); Hodgkin lymphoma (*n* = 1); optic pathway glioma (*n* = 1).

**Table 2:** Clinical profile of patients with clinical diagnosis of neurofibromatosis 1 in this study.

Neurofibromatosis 1 Diagnostic Criteria^1^	Presence of the changes/evaluated	%
*Café-au-lait* spots (>1,5 cm)	33/45	73.3
Two or more cutaneous neurofibromas	37/45	82.2
Plexiform neurofibroma^2^	17/45	37.8
Axillary freckling or freckling in inguinal regions	43/45	95.5
Optic pathway gliomas	1/45	2.2
Two or more Lisch nodules	20/23^3^	87.0
Sphenoid wing dysplasia	2/30^4^	6.7
Pseudoarthrosis	1/45	2.2
First-degree relative with neurofibromatosis 1	33/44^5^	75.0

^1^National Institute of Health Consensus Development Conference Statement: Neurofibromatosis Bethesda, 1988.

^2^Only cases confirmed by biopsy.

^3^Twenty-two patients did not attend the appointment with the ophthalmologist for personal reasons.

^4^Fifteen patients did not attend the performance of the RX for personal reasons, but none had evidence of sphenoid bone dysplasia.

^5^One person was adopted and was unaware of this information.

**Table 3:** *Bsm*I (A/G) and *Fok*I (C/T) genotypic and allelic frequencies in neurofibromatosis 1 patients and in unaffected controls.

	Patients (%) *N* = 45	Controls (%) *N* = 150	*p* value
*Bsm*I			0.284^1^
AA	4 (8.9)	23 (15.3)
AG	27 (60.0)	71 (47.3)
GG	14 (31.1)	56 (37.3)
*Bsm*I			>0.999^2^
Allele A	35 (38.9)	117 (39.0)
Allele G	55 (61.1)	183 (61.0)
*Fok*I			0.430^1^
CC	14 (31.1)	63 (42.0)
CT	26 (57.8)	73 (48.7)
TT	5 (11.1)	14 (9.3)
*Fok*I			0.314^2^
Allele C	54 (60.0)	199 (66.3)
Allele T	36 (40.0)	101 (33.7)

^1^Chi-square test; ^2^Fisher's exact test.

**Table 4:** *VDR* polymorphisms and vitamin D levels in NF1 patients.

Genotype	25(OH)D (ng/mL)		*p* value
	<30	≥30	
*Bsm*I			0.8875^1^
AA (*n* = 4)	3 (9.7)	1 (7.1)
AG (*n* = 27)	19 (61.3)	8 (57.1)
GG (*n* = 14)	9 (29.0)	5 (35.7)
*Fok*I			>0.999^1^
CC (*n* = 14)	10 (32.3)	4 (28.6)
CT (*n* = 26)	18 (58.1)	8 (57.1)
TT (*n* = 5)	3 (9.7)	2 (14.3)

^1^Fisher's exact test based on 10000 sampled tables with starting seed 2000000.

25(OH)D: 25-hydroxy-vitamin D.

**Table 5:** Studies that assessed 25(OH)D levels in patients diagnosed with NF1.

Author	Country	Method for dosing 25(OH)D levels^*∗*^	Study design	Mean age, years (cases: controls)	Number (female/male rate)	25(OH)D levels	Results (*p* value)
Lammert et al., 2006 [14]	Germany	CLBPA: autumn or winter	Case-control	40.3 : 36	55 cases (33 : 22), 58 controls (38 : 20)	Mean: 15.7 ng/mL (cases): 35.5 ng/mL (controls)	Lower 25(OH)D levels in NF1 patients (*p* < 0.0001) and inverse correlation with the number of neurofibromas (*p* < 0.0003)

Brunetti-Pierri et al., 2008 [9]	USA	Chromatography	Descriptive	13.5 (cases)	16 cases with osteoporosis or osteopenia	Mean: 20.6 ng/mL (cases)	—

Tucker et al., 2009 [10]	Germany	Technical unreported: winter and summer	Case-control	Men 43.4 and women 42.1 (cases)	72 cases (43 : 29)	<20 ng/mL in 56% (cases)	Lower 25(OH)D levels in NF1 patients (*p* < 0.001)

Seitz et al., 2010 [11]	Germany	RIA: autumn, winter, and spring	Case-control	44.36 : 46.97	14 cases (9 : 5), 42 controls (27 : 15)	Range: 5–23 ng/mL (cases): 13–46 ng/mL (controls)	Lower 25(OH)D levels in NF1 patients (<0.05)

Stevenson et al., 2011 [15]	USA	CLIA	Case-control	9.3 (controls)	109 cases (50 : 59), 218 controls	Mean: 31.76 ng/mL (cases): 33.79 ng/mL (controls)	Lower 25(OH)D levels in NF1 patients (0.0129)

Petramala et al., 2012 [12]	Italy	RIA: autumn and winter	Case-control	41.1 : 44.3	70 cases, 60 controls	Mean: 21.8 ng/mL (cases): 32.9 ng/mL (controls)	Lower 25(OH)D levels in NF1 patients (<0.01)

Hockett et al., 2013 [16]	United Kingdom	Chromatography	Case-control	11.8 : 11.5	15 cases (10 : 5), 15 controls (8 : 7)	Mean: 15.6 (cases) 16.6 (controls)	25(OH)D levels were not significantly different between groups; overall mean in total population was within deficient range

NF: neurofibromas; 25(OH)D: 25-hydroxy-vitamin D; CLBPA: chemiluminescence binding protein assay; RIA: radioimmunoassay; CLIA: chemiluminescence intra-assay.

^*∗*^Levels and cutoff values may vary according to the method used in the dosage.
